# Introductory Addresses

**Published:** 1897-10-09

**Authors:** 


### Introductory Addresses.

### St. Mary's Hospital.

The introductory address was delivered by Dr. William Gow, who insisted on the great importance of students learning their work thoroughly, for although the details of anatomy and physiology might in part escape the memory, the knowledge acquired in youth must be so assimilated as to last the whole life. He pointed out also the great necessity of learning well and practising thoroughly the technique of their art. In the old days surgery was regarded as a handicraft, but to-day the student was so overwhelmed with ologies of all kinds that he was apt to forget that it was a handicraft still, and so pass out into the world to practise his profession often entirely ignorant of how to tie a surgical knot or how to write a prescription. The study of surgical and medical technique might not count for much in the examination-room, but was of fundamental importance; and one of the foremost duties of the student was to learn how to do things in the best possible way while he had at hand the enormous opportunities for practice which a great hospital afforded. It was only by constant practice that this sort of training could be brought to perfection, and if not acquired while at the hospital, the student might never again have a chance of rectifying the omission.

He objected to the modern system of taking up the various subjects for the final examination separately, thinking that it tended to encourage cramming, and to throw into the shade the very close relationship which existed between all the branches of medical study.

### King's College.

After alluding to recent changes in the staff, and especially to the appointment of Professor Hughes, of University College, Cardiff, to the chair of anatomy, the Principal (the Rev. Dr. Robertson) drew attention to the improvements which had been carried out in the anatomical rooms of the college during the vacation. He strongly advised all students who could do so to proceed to London medical degrees. The prospect of a substantial redress of the inequality under which the London medical student laboured as a candidate for a degree, as compared with students at the greater provincial schools, was at present once again postponed. As a set-off against any such attraction to the provincial colleges as was offered by the greater accessibility of an ordinary degree to the average medical student at such places, the London medical schools enjoyed in their hospitals a field of clinical work superior to anything that a provincial school could offer. Perhaps this very fact in one sense made the existing anomaly all the more glaring, but in any case the prospect of a Bill being carried into law which should decisively remedy this hardship appeared at the present time somewhat remote.

### St. George's Hospital.

Dr. Patrick Manson, in his introductory lecture to which we have alluded in another column, devoted himself chiefly to the subject of tropical diseases, and urged the necessity of more systematic teaching of the subject, especially for such as proposed to practise in warm climates. What, he said, does the student and future tropical practitioner actually know about malaria when he is stamped as qualified to practise his profession? He has heard of the malaria germ, but has he seen it, could he recognise it? Has he been taught to find it for himself, to make use of its presence or absence in the blood as an infallible means of diagnosis? What would an examiner nowadays do with a student who could not recognise and demonstrate the tubercle bacillus? He would pluck him. And I, said Dr. Manson, if I were an examiner, and found that a student intending by-and-by to practise in the tropics, could not recognise and demonstrate the malaria parasite, I would do the same.

By instituting a lectureship on tropical medicine, St. George's had done its share in encouraging a necessary reform in medical teaching. Other schools were following the lead; but, be the schools ever so willing to second each other, without the co-operation of the General Medical Council they could do but little.

### Charing Cross Hospital.

Dr. William Carter, Professor of Materia Medica, University College, Liverpool, drew attention to the continued expansiveness of medicine, its tendency to adopt new ideas and spread into new fields. One of its chief hindrances was the exercise of authority. Of external authority there happily was but little, the profession being left to work out its own destiny. Internally, however, the effects of authority were injurious in several ways, first among which was that exercised by the dogmas uttered from time to time by its great men. Medicine appropriated the discoveries of many sciences, and must, therefore, advance with them. Dogmas should have no place in it. The statements that fever was incurable, and that Potts' curvature of the spine was incapable of sudden reduction, were examples of such dogma, which, although apparently true for centuries, were not now true, and served only to trammel the mind in its efforts to advance.

### University College, London.

The introductory address was given by Mr. Raymond Johnson, F.R.C.S., who insisted upon the importance of a medical man being in the highest sense a man of the world, and urged the students not to allow their studies so entirely to occupy their minds that the acquirement of general knowledge should be neglected. He spoke of the great advantage of outdoor exercise and of a well-balanced enthusiasm in the different forms of healthy relaxation, and in conclusion he traced the history of University College Hospital from its foundation in 1828, as a small dispensary, and maintained that it could show a record of which it might well be proud.

### Middlesex Hospital.

After distributing the prizes, Lord Strathcona and Mount Royal delivered an address in which he entered into many interesting particulars about medicine and surgery in the early days of Canadian history, pointing out the uses that had been made of the products of various indigenous trees and shrubs.

### Mason College, Birmingham.

The opening address was delivered by Sir James Crichton-Browne, M.D., F.R.S, who, while pointing out that all recent progress in medicine had depended on research carried on by physical and chemical methods, endeavoured to show the limitations which would probably bar the way when the attempt was made to reduce questions dealing with morals to terms of those sciences. The mechanical principles that were first applied in anatomy to the explanation of the construction and movement of bones and muscles had been carried by the physiologist into every organ and tissue of the body; and, at the same time, the aid of chemistry and electricity has been invoked to drive back, step by step, and, if possible, to banish altogether that vitalism which was at one time all but supreme in the domain of animal physiology. And now, not content with his corporeal conquests, the physiologist was pushing his mechanical methods into the realms of psychology. and was seeking by means of them to investigate the *data* of consciousness. Having disentangled to a large extent the paths of nervous impulses of various kinds, and their halting points and goals in nerve cells, he was now eager to catch ideas on the wing, and to examine them in the usual manner. The revelations of comparative anatomy, and the brilliant discoveries that we owe to experiments on animals, had assuredly inclined us to take an animal view of human nature. Yet, notwithstanding all homologies and resemblances, there was a great gulf fixed between human and animal nature, and man, the breviary of all bygone evolution, stood pre-eminent in form, structure, and intelligence, and above all in the possession of a moral sense. It was not only by the complexity of his brain and its tissues that man was separated from the rest of creation, so much as by what are incorrectly termed the functions of his brain, by his mental faculties, his reason, and his intelligence. Such "functions of the brain" certainly could not hold the same relation to that organ that movement did to the muscles, or bile to the liver. Nothing could be derived from motion but another motion, nothing from mental process but another mental process; and thus the facts of consciousness could never be explained by molecular changes in the brain, and all we could do was to fall back upon an hypothesis of psycophysical parallelism, which assumes concomitant variations in brain and mind. There was a physical universe of which only a fragment was known to us. There was a psychical universe, in one corner of which we lived and moved and had our being, and we might imagine these to touch at certain points. But whatever imagery we used in our endeavour to explain the matter we must hold fast to the truth that mind and matter are distinct essences, irreconcilable in their nature, though mysteriously accordant in their operations; that only in the elementary processes of mind, made up of sensory and motor elements, has correspondence with physical changes in the brain been traced out; and that we are still without any cerebral explanation of those higher powers of mind which exalt man above the animal creation.

### The Yorkshire College, Leeds.

In his opening address Mr. T. R. Jessop gave many interesting details in regard to the present state of surgery in Russia, and highly eulogised the Russian hospitals and cliniques, speaking of them as being far in advance of our own. Of the recently completed Moscow cliniques it would be difficult to speak in adequate terms. Each building was a complete hospital, with its own lecture-room, laboratory, professor's room, &c., and in those requiring it there was provided a suite of operating-rooms which might well serve as models for any hospital. Each building was adapted for a special purpose, for dealing, namely, with surgical or medical cases, children's diseases, ophthalmic, contagious, nervous, nasal, and aural affections, and so on. And all this had been done for the sole purpose of educating medical students, of providing the country with competent medical men. Thus had Russia set an example to the world, in that she recognised it as a duty of the State not only to provide hospital treatment for the poor, as was done in several other countries, but also to secure that the people should be supplied with thoroughly-trained medical men. After referring to the excellent surgical work he had seen in some of the Russian hospitals, Mr. Jessop related how, on his return to England, he had taken the opportunity of visiting three of the largest, oldest, and best reputed metropolitan hospitals, in order to see how far they had conformed to the requirements of modern surgery in their operative departments, and he felt constrained to say that their condition was such as did not reflect credit on those who were responsible for upholding the standard of British surgery. In London Lister's teaching had not yet brought forth its full fruit.

### London School of Medicine for Women.

Mr. J. Grosvenor Mackinlay, F.R.C.S.E., delivered the inaugural address at the Royal Free Hospital. He traced the growth and accumulative strength of the medical movement among women through the troublous times of its initial stages to the recent rebuff met with at Cambridge, and the refusal of the Royal College of Surgeons to grant diplomas. He advised the students to show the authorities what they could achieve without their help and with that of Newnham and Girton. The speaker characterised such policy as shortsighted; the fees in consequence were diverted into the more liberal-spirited academies and colleges of Scotland, Ireland, Belgium, and Sweden. Mr. Mackinlay, in alluding to women's medical work in her Majesty's vast dominions laid particular stress on that carried on in our Indian provinces, the principal requisite for pursuing this being good health.

### University College, Sheffield.

Dr. Pye Smith, F.R.S., after congratulating Sheffield on the opening of the new college and its admission to the Victoria University, and describing the growth of colleges in various parts of the kingdom, said that the framework of the higher education might now be considered complete; what was needed was endowments on a scale worthy of the wealthiest country in the world, to provide not only professors, demonstrators, and tutors, but also bursaries and exhibitions for poor students, libraries, museums, and laboratories.

